# Supplementary material for: survextrap: a package for flexible and transparent survival extrapolation
Source: BMC Med Res Methodol. 2023 Nov 29;23:282. doi: 10.1186/s12874-023-02094-1 (PMC10685663; doi:10.1186/s12874-023-02094-1)
Supplement: Supplementary file 1 — Additional file 1: Appendices. [file 12874_2023_2094_MOESM1_ESM.pdf]

RESEARCH

survextrap: a package for flexible and transparent survival extrapolation. Appendices

Christopher H. Jackson

Correspondence:
chris.jackson@mrc-bsu.cam.ac.uk
MRC Biostatistics Unit, University of Cambridge, Cambridge, UK

Appendix

(A) Rationale for using the M-spline model

An advantage of generalised additive models such as splines, compared to other families of flexible models that have been used for survival analysis, e.g. those based on Gaussian processes [19] or Dirichlet processes [16], is that they express a flexible function in an easily-computable form, as a linear function of basis terms. In the M-spline model, both the hazard and the cumulative hazard, required to compute the likelihood, are defined as linear functions of basis terms.

Many other kinds of spline or basis models are available, however. The model of Royston and Parmar [38], which defines the log cumulative hazard as a natural cubic spline function of log time, is often used for survival analysis, but this model does not inherently impose the required constraint that the cumulative hazard is an increasing function of time. To fit the model to data by maximum likelihood, the constraint is imposed while fitting, by rejecting proposed combinations of parameter values that are found to give decreasing cumulative hazards. However, this approach is problematic for a Bayesian model, which requires a prior distribution specified in advance of estimation. The prior should represent knowledge about plausible parameter values, and it is unclear how a prior that excludes invalid combinations of parameters would be defined in advance.

This problem would be avoided if the spline model were placed on the log *hazard* [as in 14], which does not require a constraint to be positive or increasing. However, expensive numerical integration would generally then be required to compute the cumulative hazard. The advantage of the M-spline is that it can be placed directly on the hazard, as it is designed to represent a non-negative function, and it integrates analytically so that the cumulative hazard can be computed easily.

(B) Differences from the model of Guyot et al (2017)

The model implemented in this paper for survival extrapolation with external data is similar in principle to the model used by Guyot et al. [22]. Both are spline-based, flexible parametric models. Both models are applied to combinations of clinical trial, registry and population data, using Bayesian evidence synthesis. However, our variant of this model has several advantages.

- In this paper, an M-spline basis is used, rather than the natural cubic spline [38] basis. As discussed in Appendix section A, the M-spline is equally computationally efficient, but has the advantage that the set of valid parameters for a survival model can be defined explicitly. This allows substantively-informed priors to be placed on these parameters (Section 2.5). Furthermore,

the `survextrap` package provides guidance and tools to determine these priors, given judgements about typical hazards, and variations in these hazards, expressed on an interpretable scale.

- In [22], the flexibility of the spline is defined by the number of knots. The optimal number of knots is chosen on the basis of statistical fit (measured by DIC). This paper takes a slightly more sophisticated approach. A model with a large number of knots is used. The parameters of this model are then "penalised" or "shrunk" in the direction of a simple model, through a hierarchical prior (Section 2.2). The extent of penalisation is defined by the parameter σ. This parameter is given a prior, that is updated to a posterior given the data. Hence the flexibility of the final model is determined by the number of knots, the prior and the data, with larger datasets generally encouraging more flexible models. The intention of penalisation is to mitigate against overfitting, and by estimating σ, the appropriate model flexibility is determined by the data. However, our procedure still involves choices: the initial number of knots and the prior. The statistical fit of models with different choices can be assessed by a criterion (LOOIC) which is similar to DIC (Section 2.5). Future work will involve simulation studies to assess whether the default choice in the package is reasonable in a range of realistic applications.
- The model presented here includes a nonproportional hazards model, which allows the hazard ratio for a predictor to be an arbitrarily-flexible function of time, estimated from data. While the cubic spline model can be extended to include nonproportional hazards [38], this has not previously been applied in a Bayesian evidence synthesis context, as far as I am aware.
- The model presented here includes the option of a mixture cure formulation (Section 4.6).
- Both this paper and [22] employ Markov Chain Monte Carlo (MCMC) algorithms to estimate the posterior distribution. The specific form of MCMC used here is the Hamiltonian Monte Carlo sampler in Stan (as also used for survival extrapolation in Chaudhary et al. [10]), while Guyot et al. [22] used variants of the Gibbs sampler in WinBUGS. The main advantage of Hamiltonian Monte Carlo is that it makes use of the gradients of the posterior density, which substantially reduces autocorrelation between successive samples, leading to faster convergence and more efficient estimation [9]. The problems with choice of initial values and parameter identifiability reported in Appendix E of [22] were not encounted in this paper. The only computational problem was the occasional occurence of "divergent transitions" [42], which was alleviated by reducing the initial number of knots.

However, the clearest advantage of the method in this paper over the method of Guyot et al. [22] is the vastly simpler software interface.

- For the method in [22] users must write code in the BUGS language to implement their choice of model. The example model presented in their Appendix D has over 100 lines of BUGS code, which the user must adapt for each new model and application. Additionally, the computation of the restricted mean survival time involves adding a new "module" to the standard version of WinBUGS by using Component Pascal code that must be compiled and installed separately.

- In `survextrap`, a model is fitted with a single command, and a further single command can be used to extract an output of interest from this (see Appendix (D)).

(C) M-spline construction

As defined in Section 2.1, we model a hazard function as

$$h(t) = \eta \sum_{i=1}^{n} p_i b_i(t)$$

where $\eta > 0$ and $p_i : i = 1, \ldots, n, \sum_i p_i = 1$ are parameters to be estimated, and the $b_i(t)$ are deterministic functions of time t, known as "basis functions". The basis functions are defined using an extension of the standard "M-spline" basis.

Standard M-spline basis The standard M-spline basis, as described by Ramsay [37] after Curry and Schoenberg [15], is designed to build flexible functions $h(t)$ of a variable t, where t is in a finite interval $[L, U]$. To construct this, a grid of points $t_1, \ldots, t_{n+k}$ on this interval is defined, where

- $t_1 = \ldots = t_k$ is the "lower boundary" L,
- t_{k+1} up to t_n comprise the "internal knots",
- $t_{n+1} = \ldots = t_{n+k}$ is U, the "upper boundary" knot,
- k is the "order" of the basis. This governs the degree d of the polynomials that are used to define the basis. $d = k - 1$, so that if $k = 2$, then the basis functions will be piecewise linear ($d = 1$), and if $k = 4, d = 3$, the functions will be built from cubic polynomials.

Given an order k, the ith basis term $b_i(t|k)$ for $i = 1, \ldots, n$ is defined recursively as follows. Firstly the $b_i(t|k)$ are defined for $k = 1$ as

$$b_i(t|k=1) = 1/(t_{i+1} - t_i) \quad \begin{array}{l} \text{if } t \text{ is between the knots } t_i, t_{i+1} \\ 0 \text{ otherwise} \end{array}$$

Then for each subsequent order k, $b_i(t|k)$ are defined in terms of the basis functions for the previous order $k - 1$, as

$$b_i(t|k) = \frac{k\left[(t - t_i)b_i(t|k-1, t) + (t_{i+k} - t)b_{i+1}(t|k-1, t)\right]}{(k-1)(t_{i+k} - t_i)}$$

For example, this defines $b_i(t|k = 2)$ to be triangular functions on (t_i, t_{i+2}) with mode at t_{i+1}. Whatever the degree, $b_i(t)$ are positive in (t_i, t_{i+k}), zero elsewhere, and integrate to 1 (see, e.g. Figure 1).

If $p_i = \frac{t_{i+k} - t_i}{k(U-L)}$ for $i = 1, \ldots, n$, then $\sum_{i=1}^{n} p_i b_i(t)$ is constant with time, and $p_i b_i(t)$ becomes a basis function for a "B-spline" [37], which is used for modelling unrestricted functions.

Extension to model hazard functions Here this standard M-spline basis is extended to define a spline that can represent hazard functions $h(t)$ for time-to-event analysis. The standard basis already satisfies the requirement that $h(t) \geq 0$, but we also

require $h(t)$ to be defined for any time $t \geq 0$, rather than just a finite interval. To achieve this:

1 the lower boundary is fixed to $L = 0$,

2 the value of $h(t)$ for all $t > U$ is taken to be constant, and defined by the value $h(U)$.

The "knots" comprise the internal knots and the upper boundary U. These are chosen by the user to give an appropriately-flexible function for their application.

The spline definition for the Royston and Parmar [38] model assumes that the log cumulative hazard is linear for times greater than the highest knot, as in the Weibull model. When fitted to data observed up to U, this has the effect of extrapolating the hazard *trend* observed in the later data. In contrast, the M-spline model here makes a more conservative assumption that the *level* of the hazard after U is extrapolated from the later data.

Further extension for smoothness at the boundary We can also improve the function's smoothness around the upper boundary U by restricting the derivative and second derivative at U to both be zero. Without loss of generality, suppose that the scale parameter $\eta = 1$, so that $h(t) = \sum_i p_i b_i(t)$. To deduce the simplified basis, we first note that, for degree $d = 3$, the $h(t)$ and its derivatives at the upper boundary U take the simple form

$$\begin{aligned} h(U) &= p_n b_n(U) \\ h'(U) &= p_{n-1} b'_{n-1}(U) + p_n b'_n(U) \\ h''(U) &= p_{n-2} b''_{n-2}(U) + p_{n-1} b''_{n-1}(U) + p_n b''_n(U) \end{aligned}$$

since the other $b_i(t)$ and their derivatives are zero at U. Therefore if $h'(U) = h''(U) = 0$, we can deduce that

$$\begin{aligned} p_n &= C/b_n(U) \\ p_{n-1} &= (C/b_n(U))(b'_n(U)/b'_{n-1}(U)) \\ p_{n-2} &= (C/b_n(U))(b'_n(U)/b'_{n-1}(U))b''_{n-1}(U) + (C/b_n(U))b''_n(U) \end{aligned}$$

where $h(U) = C$. This allows us to define a simpler basis with two fewer terms,

$$h(t) = \sum_{i=1}^{n-2} p^*_i b^*_i(t),$$

by replacing the final three terms of the original basis $p_{n-2}b_{n-2}(t)+p_{n-1}b_{n-1}(t)+p_n b_n(t)$ with a single term $p^*_{n-2}b^*_{n-2}(t)$. In this new term,

- the coefficient is $p^*_{n-2} = C$, easily interpreted as the hazard value at U, and
- $b^*_{n-2}(t)$ is computed as a function of the original basis terms $b_n(t)$, $b_{n-1}(t)$, $b_{n-2}(t)$ and their values, derivatives and second derivatives at U.

(D) Example of basic use of `survextrap`

The dataset `colons` gives survival or censoring times of 191 patients from a trial of two chemotherapy regimes for colon cancer: levamisole (`"Lev+"`) and levamisole combined with fluorouracil (`"Lev+5FU"`), compared to an observation-only control group (`"Obs"`). Suppose there are also external data, giving 50 survivors to 10 years out of 100 people alive at 5 years, and 40 survivors to 15 years out of 100 alive at 10 years.

```
extdat <- data.frame(start = c(5, 10), stop =  c(10, 15),
                     n = c(100, 100), r = c(50, 40), rx = "Obs")
```

We fit a proportional hazards model to the individual and external data jointly. The prior for the log hazard ratio is changed from its default, and assumed to apply to both treatment groups.

```
library(survextrap)
npc_mod <- survextrap(Surv(years, status) ~ rx, data=colons,
                      prior_loghr = p_normal(0, 1.5),
                      external = extdat)
```

Posterior summaries of the hazard ratios for each chemotherapy versus control, restricted mean survival at 5 years, and survival probabilities at 5 and 10 years, are produced. These are the median and 95% credible intervals by default, but any summary can be produced. The outputs are all data frames, with one row per quantity of interest, and columns that define the quantity and give different posterior summaries for it. This is intended to obey “tidy data” principles, hence to facilitate further data processing and plotting, e.g. with the `ggplot2` package. 100 iterations `niter` from the posterior are used for a quicker but rougher summary — this should be increased if more precision is needed.

```
summary(npc_mod) %>%
    filter(variable=="hr") %>%
    select(term, median, lower, upper)
```

```
## # A tibble: 2 x 4
##   term      median lower upper
##   <chr>      <dbl> <dbl> <dbl>
## 1 rxLev      0.719 0.427 1.14
## 2 rxLev+5FU  0.542 0.319 0.926
```

```
rmst(npc_mod, t=3, niter=100)
```

```
##        rx variable t median 2.5% 97.5%
## 1     Obs     rmst 3   2.02 1.82  2.19
## 2     Lev     rmst 3   2.27 2.00  2.47
## 3 Lev+5FU     rmst 3   2.42 2.18  2.61
```

```
survival(npc_mod, t=c(5,10), niter=100)
```

```
##          rx  t median lower upper
## 1       Obs  5  0.366 0.263 0.448
## 1.1     Obs 10  0.165 0.103 0.211
## 2       Lev  5  0.480 0.347 0.616
```

```
## 2.1     Lev 10  0.276 0.154 0.422
## 3   Lev+5FU  5  0.592 0.434 0.700
## 3.1 Lev+5FU 10  0.391 0.218 0.541
```

The default plot method gives the estimated survival and hazard, and optionally also uncertainty intervals around these.

```
plot(npc_mod, xlab="Years", niter=100)
```

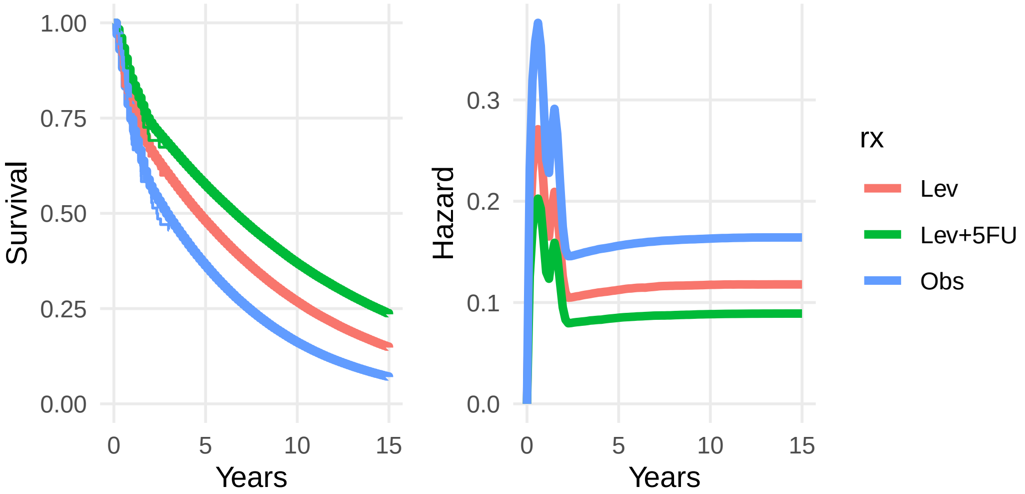

Examples of more advanced usage, and thorough documentation for all functions and features, are available at `https://chjackson.github.io/survextrap`.
