# Supplementary material for: survextrap: a package for flexible and transparent survival extrapolation
Source: BMC Med Res Methodol. 2023 Nov 29;23:282. doi: 10.1186/s12874-023-02094-1 (PMC10685663; doi:10.1186/s12874-023-02094-1)
Supplement: Supplementary file 2 — Additional file 2: Further details and reproducible code for the cetuximab case study [file 12874_2023_2094_MOESM2_ESM.html]

Case study of using survextrap: cetuximab for head and neck cancer


Code 

- Show All Code
- Hide All Code

# Case study of using survextrap: cetuximab for head and neck cancer

#### Christopher Jackson chris.jackson@mrc-bsu.cam.ac.uk

#### 2023-10-24

This article is an in-depth demonstration of how
`survextrap` could be used in a realistic application to a
health technology evaluation. A range of data sources and analysis
choices are explored, and publication-style plots and tables are
produced.

A more formal account of this analysis is given in the preprint paper about the package and
method. This article also serves the purpose of providing the code that
was used in the paper, making the analysis reproducible and explaining
how the code is used.

A more casual, quick tour of the package’s features is given in the
`survextrap` package vignette called examples.html, and the methods are explained in
detail in methods.html.

# Simple data summary

The data are from an analysis of survival in head and neck cancer,
previously conducted by Guyot
et al.. The `survextrap` package provides the data as
`cetux`, `cetux_seer` and `cetux_bh`.
`cetux` is data from a clinical trial of cetuximab and
radiotherapy, compared to a control group who only received
radiotherapy. `cetux_seer` contain matched data from a USA
cancer registry, and `cetux_bh` describes comparable survival
for the general population of the USA. See `help("cetux")`
and Guyot
et al. for more information.

We first load the required packages, plot a Kaplan-Meier curve of the
individual trial data, and create subsets of the data by treatment
group, for use in some of the later analyses.

```
library(survextrap)
library(ggplot2)
library(dplyr)
library(viridis) # for colour palettes
survminer::ggsurvplot(survfit(Surv(years, d) ~ treat, data=cetux)) + xlab("Years")
```

```
control <- cetux[cetux$treat=="Control",]
cetuximab <- cetux[cetux$treat=="Cetuximab",]
```

# Spline specification

First we set up an M-spline, specifying only that we expect 6 spline
coefficients to be sufficient to describe hazard variations, and we want
to allow hazards to change up to 20 years. The resulting default spline
knots are saved in the `mspline` object here. The default
knots are based on quantiles of the event times in the data (which is
why we supply the data in this command).

```
mspline <- mspline_spec(Surv(years, d) ~ 1, data=cetux, df=6, add_knots=20)
```

# Choice of priors

For transparency here, all priors are specified explicitly, rather
than relying on the package defaults. The parameters of spline models
hard to interpret, but we can determine suitable priors by translating
them to quantities that are easier to interpret. This will sometimes
require simulation. Note also that knowledge of the spline knots is
necessary to do some of these calculations, which is why we set up the
`mspline` object in advance of setting up the priors. See the
linked function documentation for more details about how these functions
work.

#### Hazard scale parameter \(\eta\).

Since patients in the trial have a median age of 57 (range 34 to 83),
the prior for \(\eta\) is calibrated to
imply a prior mean survival of 25 years after diagnosis, but with a
variance chosen so that mean survival could be as high as 100 years
after diagnosis. The function `p_meansurv()` translates a
prior median and upper 95% credible limit for mean survival to an
appropriate normal distribution for the log hazard scale \(\eta\). Note that this distribution
represents uncertainty about the population mean, not individual
variability (which should be broader).

Conversely, the `prior_haz_const()` function translates a
normal prior for \(\eta\) to the
corresponding beliefs about survival. We use this to check that the
lower limit of the distribution derived by `p_meansurv()` is
sensible.

```
prior_hscale <- p_meansurv(median=25, upper=100, mspline=mspline)
prior_haz_const(mspline, prior_hscale = prior_hscale)
```

```
##        haz   mean
## 2.5%  0.01   6.25
## 50%   0.04  25.00
## 97.5% 0.16 100.00
```

#### Hazard variability parameter \(\sigma\).

The prior for \(\sigma\) is chosen
so that the highest hazard values over the 20 year horizon (90%
quantile) are expected to be about \(\rho=2\) times the lowest values (10%
quantile), but with a vague 95% credible interval for \(\rho\) of between about 1 and 16. The
function `prior_haz_sd()` uses simulation to estimate the
beliefs implied by a particular Gamma prior for \(\sigma\) (jointly with the prior specified
for the hazard scale).

The choice of Gamma(2,5) here was arrived at through trial-and-error,
until the distribution for \(\rho\)
(the quantity `hr` returned by `prior_haz_sd()`)
matched our prior judgement. Note that repeated simulations will give
slightly different results unless the seed is set.

```
set.seed(1)
prior_hsd <- p_gamma(2, 5)
prior_haz_sd(mspline = mspline,
             prior_hsd = prior_hsd,
             prior_hscale = prior_hscale)
```

```
##             sd_haz    sd_mean        hr
## 2.5%  0.0002520058   1.374872  1.060858
## 50%   0.0036770673  17.146610  1.855044
## 97.5% 0.0371823461 303.313735 15.759463
```

#### Hazard ratio for treatment effects

The prior for the treatment effect is weak, and designed only to rule
out extremely implausible values, with a prior median of 1 and upper 95%
credible limit of 50 for the hazard ratio. The function
`p_hr()` translates this information to a normal prior for
the log hazard ratio. The function `prior_hr()` goes in the
other direction, and summarises the prior for the hazard ratio implied
by a particular normal distribution on the log scale (in the special
format returned by `p_hr()`, or alternatively by the function
`p_normal()`).

```
prior_loghr <- p_hr(median=1, upper=50)
prior_hr(prior_loghr)
```

```
##  2.5%   50% 97.5% 
##  0.02  1.00 50.00
```

#### Hazard ratio variability parameter \(\tau\) in non-proportional hazards model

In a similar way, we can calibrate the Gamma prior for the parameter
\(\tau\), which is only used in
non-proportional hazards models. This governs the size of departure from
proportional hazards, i.e. the variability in the hazard ratio over
time. \(\tau\) itself does not have a
direct interpretation, but we can simulate hazard curves under different
treatment groups, and compute the ratio \(\rho\) between the “highest” (90% quantile)
and “lowest” (10% quantile) hazard ratio over time. If hazards are
proportional, then \(\rho=1\).

Here a Gamma(2,3) prior is used, which represents a judgement that
the hazard ratio could vary up to 7-fold at different points over 20
years.

```
set.seed(1)
prior_hrsd <- p_gamma(2, 3)
prior_hr_sd(mspline = mspline,                              
            prior_hsd = prior_hsd,
            prior_loghr = prior_loghr,
            prior_hscale = prior_hscale,
            prior_hrsd = prior_hrsd,
            nonprop = ~treat,
            formula = ~treat,
            newdata = data.frame(treat=1), 
            newdata0 = data.frame(treat=0))
```

```
##              sd_hr       hrr
## 2.5%   0.004380155  1.986133
## 50%    0.392423647  4.703221
## 97.5% 26.246758325 31.972443
```

# Notes on model computation

By default, `survextrap` fits models by MCMC sampling.
This can be slow, and with the default settings takes 2 minutes per run.
For model development, we can reduce the number of MCMC chains and
iterations by setting `chains` and `iter` when
calling `survextrap`. These default to 4 and 2000
respectively in the `rstan` package that
`survextrap` uses for computation.

An approximation to full MCMC sampling can also be specified by
calling `survextrap` with `fit_method="opt"`: this
runs instantly and obtains the posterior mode exactly, but crudely
approximates other posterior summaries. This is very useful for
development.

When we are ready to produce the “final” results, we can switch back
to MCMC with the default number of chains and iterations.

```
options(mc.cores = 2) # CRAN check limits to 2 cores
# options(mc.cores = parallel::detectCores())
chains <- 4; iter <- 2000
```

The `rhat` convergence diagnostic shown when printing the
results of a `survextrap` fit should be checked - this should
be close to 1 if the MCMC fit has converged.

Occasionally the fitting may report “divergent transitions”. This is
a limitation of the sampling algorithm, and can happen when the
posterior distribution is awkward to sample from. See the Stan documentation for
technical details. If there are only one or two divergent transitions,
the message is probably safe to ignore, but in any case the results
should be examined to ensure that they make sense. With lots of
divergent transitions, it is safer to simplify the model, e.g. by using
stronger priors or fewer spline knots.

# Models for the trial data alone

## Single treatment group

First we fit two models for the control group trial data: one
(`mod_con`) allowing hazard variations up to 20 years, and
another (`mod_con5`) assuming a constant hazard after 5
years. `mod_con5` is implemented by leaving out the
`mspline` argument to `survextrap`, in which case
the final spline knot is set to the end of the data. All models in
`survextrap` assume a constant hazard after the final spline
knot.

```
mod_con <- survextrap(Surv(years, d) ~ 1, data=control, mspline=mspline,
                      chains=chains, iter=iter,
                      prior_hscale=prior_hscale, prior_hsd = prior_hsd)
mod_con5 <- survextrap(Surv(years, d) ~ 1, data=control, 
                       chains=chains, iter=iter, 
                       prior_hscale=prior_hscale, prior_hsd = prior_hsd)
```

A plot comparing the survival and hazard curves from these models is
then built. We use the `survival()` and `hazard()`
functions to extract survival and hazard estimates, and combine these to
give a dataset that can be plotted with `ggplot()`.

```
surv_const <- survival(mod_con5, tmax=20) %>% mutate(model="Constant hazard")
surv_ipd <- survival(mod_con, tmax=20) %>% mutate(model="Varying hazard")
surv_single <- rbind(surv_const, surv_ipd)
haz_const <- hazard(mod_con5, tmax=20) %>% mutate(model="Constant hazard")
haz_ipd <- hazard(mod_con, tmax=20) %>% mutate(model="Varying hazard")
haz_single <- rbind(haz_const, haz_ipd)

ps <- ggplot(surv_single, aes(x=t, y=median, 
                        group=model, col=model, fill=model)) + 
    geom_ribbon(aes(ymin=lower, ymax=upper), alpha=0.2, colour=NA) +
    geom_line(lwd=1.3) + 
    geom_step(data=mod_con$km, aes(x=time, y=surv), lwd=1.3,
              inherit.aes = FALSE) +
    theme_minimal() + 
    theme(panel.grid.major = element_blank(), panel.grid.minor = element_blank()) +
    xlab("Years after diagnosis") + 
    ylab("Survival probability") +
    scale_color_viridis(discrete=TRUE) + 
    scale_fill_viridis(discrete=TRUE) + 
    theme(legend.position = c(0.6, 0.8)) + labs(col=NULL, fill=NULL) +
    theme(panel.grid.major = element_blank(), panel.grid.minor = element_blank()) +
    geom_vline(xintercept = max(control$years)) +
    geom_vline(xintercept = mod_con5$mspline$iknots, col="gray80", lty=2)

ph <- ggplot(haz_single, aes(x=t, y=median, 
                        group=model, col=model, fill=model)) + 
    geom_ribbon(aes(ymin=lower, ymax=upper), alpha=0.2, colour=NA) +
    geom_line(lwd=1.3) + 
    theme_minimal() + xlab("Years after diagnosis") + ylab("Hazard") +
    theme(panel.grid.major = element_blank(), panel.grid.minor = element_blank()) +
    scale_color_viridis(discrete=TRUE) + 
    scale_fill_viridis(discrete=TRUE) + 
    geom_vline(xintercept = max(control$years)) +
    geom_vline(xintercept = mod_con5$mspline$iknots, col="gray80", lty=2) + 
    theme(legend.position = "none")

grid::grid.newpage()
grid::grid.draw(cbind(ggplotGrob(ps), 
                      ggplotGrob(ph)))
```

The plots show the posterior median and 95% credible intervals. There
are no data between 5 and 20 years, so the extrapolation of the trial
data over this period depends on the model assumption - either that the
hazard is constant, or that the hazard will change smoothly, but with an
unknown direction and extent of change. If we allow it to change, the
uncertainty is appropriately greater.

## Determining an optimal number of knots

The model seems to fit the trial data well, and the Bayesian
procedure is designed to mitigate against overfitting from having too
many spline knots. Even so, we might still want reassurance that the
spline function has an appropriate amount of complexity to describe the
data. Therefore we use LOOIC to compare a range of models with different
numbers of spline basis functions (set via the `df` argument
to `mspline_spec`).

```
dfs <- 5:12
rescomp <- as.data.frame(matrix(nrow=length(dfs), ncol=7))
for (i in seq_along(dfs)){
    msp <- mspline_spec(Surv(years, d) ~ 1, data=control, df=dfs[i])
    mod <- survextrap(Surv(years, d) ~ 1, data=control, mspline=msp,
                      chains=chains, iter=iter,
                      prior_hscale = prior_hscale, prior_hsd = prior_hsd) 
    rescompi <- cbind(list(df = dfs[i]), 
                  list(looic = mod$loo$estimates["looic","Estimate"]),
                  rmst(mod,t=5))
    rescomp[i,] <- rescompi
}
```

```
## Warning: There were 2 divergent transitions after warmup. See
## https://mc-stan.org/misc/warnings.html#divergent-transitions-after-warmup
## to find out why this is a problem and how to eliminate them.
```

```
## Warning: Examine the pairs() plot to diagnose sampling problems
```

```
## Warning: Tail Effective Samples Size (ESS) is too low, indicating posterior variances and tail quantiles may be unreliable.
## Running the chains for more iterations may help. See
## https://mc-stan.org/misc/warnings.html#tail-ess
```

```
## Warning: There were 1 divergent transitions after warmup. See
## https://mc-stan.org/misc/warnings.html#divergent-transitions-after-warmup
## to find out why this is a problem and how to eliminate them.
```

```
## Warning: Examine the pairs() plot to diagnose sampling problems
```

```
## Warning: There were 1 divergent transitions after warmup. See
## https://mc-stan.org/misc/warnings.html#divergent-transitions-after-warmup
## to find out why this is a problem and how to eliminate them.
```

```
## Warning: Examine the pairs() plot to diagnose sampling problems
```

```
names(rescomp) <- names(rescompi)
rescomp %>% 
  mutate(looic = round(looic, 1), 
         rmf = sprintf("%s (%s, %s)", round(median,2), 
                       round(`2.5%`, 2), round(`97.5%`, 2))) %>%
  select(df, looic, rmf) %>%
  knitr::kable(col.names = c("df", "LOOIC", "Restricted mean survival (5 years)"))
```

| df | LOOIC | Restricted mean survival (5 years) |
| --- | --- | --- |
| 5 | 596.6 | 2.88 (2.63, 3.13) |
| 6 | 597.5 | 2.88 (2.63, 3.14) |
| 7 | 591.1 | 2.87 (2.62, 3.14) |
| 8 | 591.5 | 2.88 (2.62, 3.13) |
| 9 | 593.7 | 2.88 (2.61, 3.14) |
| 10 | 594.0 | 2.88 (2.62, 3.15) |
| 11 | 592.3 | 2.88 (2.63, 3.15) |
| 12 | 592.7 | 2.88 (2.62, 3.14) |

The estimates of RMST within 5 years do not change within 0.1. Note
this is only one of many ad-hoc checks we could do - note we did not
change the prior for the smoothness `prior_hsd`, which also
governs the amount of smoothness of the hazard function. LOOCV is also
lower for `df=7`. In practice we might want to switch the
`df` according to what data are included, but for simplicity
of illustration in this article, we stick with `df=6` for all
models, which in general gives slightly stabler computation without
affecting the results.

Using LOOIC for model choice may result in fitted hazard functions
that wiggle a lot over time and appear to “overfit” the data - but this
may not matter if we just want to use the model for estimating
quantities averaged over time, such as restricted mean survival. LOOIC
is designed for *prediction*. If instead we want to use it for
*description* of the hazard trajectory, we might want to pick a
simpler model by setting `df` to a lower value.

## Models for the trial data with a treatment effect

We now fit a joint model for both arms of the trial data, describing
the treatment effect either through proportional hazards
(`mod_ph`) or with `survextrap`’s novel flexible
non-proportional hazards model (`mod_nph`).

```
mod_ph <- survextrap(Surv(years, d) ~ treat, data=cetux, 
                     mspline=mspline, 
                     chains=chains, iter=iter,
                     prior_hscale=prior_hscale, prior_hsd = prior_hsd,
                     prior_loghr=prior_loghr)
```

```
## Warning: There were 2 divergent transitions after warmup. See
## https://mc-stan.org/misc/warnings.html#divergent-transitions-after-warmup
## to find out why this is a problem and how to eliminate them.
```

```
## Warning: Examine the pairs() plot to diagnose sampling problems
```

```
mod_nph <- survextrap(Surv(years, d) ~ treat, data=cetux, mspline=mspline, 
                      chains=chains, iter=iter,
                      nonprop = TRUE,
                      prior_hscale=prior_hscale, prior_hsd = prior_hsd,
                      prior_loghr=prior_loghr, prior_hrsd=prior_hrsd)
```

We also fit a model for the cetuximab treatment arm alone, to couple
with the model `mod_con` for the control arm that we fitted
earlier.

```
mod_cet <- survextrap(Surv(years, d) ~ 1, data=cetuximab, mspline=mspline, 
                      chains=chains, iter=iter,
                      prior_hscale=prior_hscale, prior_hsd = prior_hsd)
```

The survival curves for these three models are now compared.

```
surv_ph <- survival(mod_ph, tmax=7) %>% mutate(Model="Proportional hazards")
surv_nph <- survival(mod_nph, tmax=7) %>% mutate(Model="Non-proportional hazards")
surv_cet <- survival(mod_cet, tmax=7) %>% 
    mutate(Model="Separate fits", treat="Cetuximab") %>% relocate(treat)
surv_con <- survival(mod_con, tmax=7) %>% 
    mutate(Model="Separate fits", treat="Control") %>% relocate(treat)
surv_trt <- rbind(surv_ph, surv_nph, surv_cet, surv_con)

ggplot(surv_trt, aes(x=t, y=median, col=Model, lty=treat)) + 
    geom_step(data=mod_ph$km, aes(x=time, y=surv, lty=treat), lwd=1.3,
              inherit.aes = FALSE) +
    geom_line(lwd=1.1) + 
    labs(lty="Treatment", col=NULL) +
    theme_minimal() +
    theme(legend.position = c(0.6, 0.8)) +
    theme(panel.grid.major = element_blank(), panel.grid.minor = element_blank()) +
    xlab("Years after diagnosis") + 
    ylab("Survival probability") +
    scale_color_viridis(discrete=TRUE) + 
    geom_vline(xintercept = max(cetux$years)) +
    geom_vline(xintercept = mod_con5$mspline$iknots, col="gray80", lty=2)
```

The results and goodness-of fit are compared between these three
models. We compare estimates of restricted mean survival for the control
group (`rmst()`), and the increase in mean survival time for
the treated group (`irmst()` ). Both of these are restricted
to the 5-year trial horizon (`t=5`).

Note that when using functions like `rmst()` or
`survival()` which extract outputs from models with
covariates, we supply `newdata` to say what covariate values
we want the outputs to be computed for. In the call to
`rmst()` we told it to compute for the control group. We left
out `newdata` in the call to `irmst()` because,
for models with one factor covariate with two levels, it will calculate,
by default, the mean for the second level minus the mean for the first
level.

```
comp_fn <- function(mod){
    rm <- rmst(mod, niter=1000, newdata=data.frame(treat="Control"), t=c(5,20)) %>% 
        rename(rm_med="median",rm_lower="2.5%", rm_upper="97.5%") %>% 
        select(t, rm_med, rm_lower, rm_upper)
    irm <- irmst(mod, niter=1000, t=c(5,20)) %>%
        rename(ir_med="median",ir_lower="2.5%", ir_upper="97.5%") %>%
        select(ir_med, ir_lower, ir_upper)
    cbind(name=deparse(substitute(mod)), rm, irm, 
          list(looic = mod$loo$estimates["looic","Estimate"]))
}

rmst_cet <- rmst(mod_cet, t=c(5,20))
rmst_con <- rmst(mod_con, t=c(5,20)) %>%
  rename(rm_med="median",rm_lower="2.5%", rm_upper="97.5%") %>% 
  select(t, rm_med, rm_lower, rm_upper)
irmst_sep <- attr(rmst_cet,"sample")[[1]] - attr(rmst_con,"sample")[[1]]
irmst_sep <- posterior::summarise_draws(irmst_sep, median,
                                        ~quantile(.x, probs=c(0.025, 0.975))) %>%
  rename(t=variable, ir_med="median",ir_lower="2.5%", ir_upper="97.5%") %>% 
  select(ir_med, ir_lower, ir_upper)
loo_sep <- mod_con$loo$estimates["looic","Estimate"] +
  mod_cet$loo$estimates["looic","Estimate"]
comp_sep <- cbind(list(name="mod_sep"), rmst_con, irmst_sep, list(looic=loo_sep))

trtcomp <- rbind(comp_fn(mod_ph), comp_fn(mod_nph), comp_sep)
trtcompf <- trtcomp %>%
  arrange(t) %>%
  mutate(rm = sprintf("%s (%s,%s)", round(rm_med,2),
                      round(rm_lower,2), round(rm_upper,2)),
         ir = sprintf("%s (%s,%s)", round(ir_med,2),
                      round(ir_lower,2), round(ir_upper,2)),
         looic = round(looic),
         model = forcats::fct_recode(name, 
                                     "(2a) Proportional hazards"="mod_ph",
                                     "(2b) Non-proportional hazards"="mod_nph",
                                     "(2c) Separate arms"="mod_sep"
         )) %>%
  select(model, t, rm, ir, looic) %>%
  tibble::remove_rownames()
knitr::kable(trtcompf, col.names=c("Model","Time horizon",
                                   "Restricted mean survival",
                                   "Increase in restricted mean survival",
                                   "LOOIC"))
```

| Model | Time horizon | Restricted mean survival | Increase in restricted mean survival | LOOIC |
| --- | --- | --- | --- | --- |
| (2a) Proportional hazards | 5 | 2.89 (2.62,3.14) | 0.31 (-0.06,0.67) | 1156 |
| (2b) Non-proportional hazards | 5 | 2.88 (2.62,3.14) | 0.31 (-0.06,0.68) | 1157 |
| (2c) Separate arms | 5 | 2.88 (2.63,3.13) | 0.36 (0,0.73) | 1160 |
| (2a) Proportional hazards | 20 | 4.97 (3.82,7.11) | 1.1 (-0.22,2.61) | 1156 |
| (2b) Non-proportional hazards | 20 | 4.98 (3.76,6.66) | 1.12 (-0.91,3.26) | 1157 |
| (2c) Separate arms | 20 | 5.11 (3.77,7.14) | 1.33 (-1.24,4.12) | 1160 |

There is very little difference between the fit of the three
models.

The following code formats the table in LaTeX format (for the
paper).

```
write.table(trtcompf, sep="  &  ", eol="\\\\\n", quote=FALSE, row.names = FALSE)
```

```
## model  &  t  &  rm  &  ir  &  looic\\
## (2a) Proportional hazards  &  5  &  2.89 (2.62,3.14)  &  0.31 (-0.06,0.67)  &  1156\\
## (2b) Non-proportional hazards  &  5  &  2.88 (2.62,3.14)  &  0.31 (-0.06,0.68)  &  1157\\
## (2c) Separate arms  &  5  &  2.88 (2.63,3.13)  &  0.36 (0,0.73)  &  1160\\
## (2a) Proportional hazards  &  20  &  4.97 (3.82,7.11)  &  1.1 (-0.22,2.61)  &  1156\\
## (2b) Non-proportional hazards  &  20  &  4.98 (3.76,6.66)  &  1.12 (-0.91,3.26)  &  1157\\
## (2c) Separate arms  &  20  &  5.11 (3.77,7.14)  &  1.33 (-1.24,4.12)  &  1160\\
```

# External data: registry data on control group

In this example, there are registry data giving counts of \(r\_j\) survivors to the next year out of
\(n\_j\) alive at year \(j\), from from \(j=5\) years onwards. This is supplied in
the `survextrap` package as `cetux_seer`.

```
head(cetux_seer)
```

```
##   start stop   r   n   treat        haz haz_upper  haz_lower
## 1     5    6 325 358 Control 0.09670780 0.1325969 0.06665828
## 2     6    7 285 308 Control 0.07761060 0.1125955 0.04927675
## 3     7    8 198 221 Control 0.10989567 0.1595617 0.06981746
## 4     8    9 143 167 Control 0.15514918 0.2239099 0.09969098
## 5     9   10 122 133 Control 0.08632808 0.1449198 0.04325704
## 6    10   11 105 117 Control 0.10821358 0.1783369 0.05615430
```

This data can be illustrated as annual hazard estimates, calculated
as \(-\log(r\_j/n\_j)\), shown as the
thin step function in the plot below.

We fit a model which assumes that people in the registry data have
the same survival as the control group in the trial. To allow for
potential variations in the hazard shape between each of the years from
6 to 25 for which registry data are reported, we add extra knots at 10,
15 and 20 years.

> **Note**: if instead there had been only one row in the
> external data, giving survivors to 25 years from those alive at 6 years,
> it would be impossible to identify variations in the hazard
> *within* the period from 6 to 25 years, so using multiple knots
> inside this period would be futile.

```
mspline_seer <- mspline_spec(Surv(years, d) ~ 1, data=cetux, df=6, 
                             add_knots=c(10,15,20))
mod_seer <- survextrap(Surv(years, d) ~ 1, data=control, 
                       external=cetux_seer, mspline=mspline_seer, 
                       chains=chains, iter=iter, 
                       prior_hscale=prior_hscale, prior_hsd = prior_hsd)
```

The results are illustrated, and compared to the model
`mod_con` for the trial data alone that allowed the hazard to
change after the trial. The registry data makes the hazard
extrapolations much more confident.

```
haz_con <- hazard(mod_con) %>%
    mutate(Model="No external data")
haz_seer <- hazard(mod_seer,tmax=30) %>% 
    mutate(Model="With registry data")

haz_plot_reg <- rbind(haz_con, haz_seer) %>% filter(t < 26)

ggplot(haz_plot_reg, aes(x=t, y=median, col=Model, fill=Model)) + 
    geom_ribbon(aes(ymin=lower, ymax=upper), alpha=0.2, colour=NA) +
    geom_line() +
    geom_step(data=cetux_seer, aes(x=start, y=haz), col="gray30", inherit.aes = FALSE) +
    geom_step(data=cetux_seer, aes(x=start, y=haz_lower), col="gray70", lty=2, inherit.aes = FALSE) +
    geom_step(data=cetux_seer, aes(x=start, y=haz_upper), col="gray70", lty=2, inherit.aes = FALSE) + 
    theme(panel.grid.major = element_blank(), panel.grid.minor = element_blank()) +
    xlab("Years after diagnosis") + 
    ylab("Hazard") +
    scale_color_viridis(discrete=TRUE) + 
    scale_fill_viridis(discrete=TRUE) + 
    geom_vline(xintercept = max(control$years))  + 
    theme(legend.position = c(0.4, 0.9), 
          legend.background=element_blank()) + 
    labs(col=NULL, fill=NULL)
```

## Knot selection for external data

The hazard seems to fit the external data, even with just one knot
designed to capture hazard trajectory changes after the trial, placed at
20 years. For some reassurance, we examine some alternative knot
specifications, to ensure that we have captured all variations that we
need in order to estimate survival up to 20 years.

Placing knots at 10,15 and 20 years gives the lowest (best) LOOIC
from these alternatives. Adding more knots is judged to be over-fitting,
which hampers predictive ability, and fewer knots do not represent all
hazard variations. The changes in the RMST estimates between these
models are small though.

```
knot_list <- list(c(10,20), 
                  c(20),
                  seq(8,20,by=2))

comp_fn <- function(mod){
  rm <- rmst(mod, niter=1000, newdata=data.frame(treat="Control"), t=c(20)) %>% 
    rename(rm_med="median",rm_lower="2.5%", rm_upper="97.5%") %>% 
    mutate(rmf = sprintf("%s (%s, %s)", round(rm_med,2), 
                         round(rm_lower,2), round(rm_upper,2))) %>%
    select(t, rmf)
  cbind(name=deparse(substitute(mod)), rm,  
        list(looic = round(mod$loo_external$estimates["looic","Estimate"])))
}

res <- vector(3, mode="list")
for (i in 1:3){
  mspex <- mspline_spec(Surv(years, d) ~ treat, data=cetux, df=6, 
                         add_knots = knot_list[[i]])
  mod <- survextrap(Surv(years, d) ~ 1, data=control, 
                    external=cetux_seer, mspline=mspex, 
                    chains=chains, iter=iter, 
                    prior_hscale=prior_hscale, prior_hsd = prior_hsd)
  res[[i]] <- comp_fn(mod)
}
```

```
## Warning: There were 1 divergent transitions after warmup. See
## https://mc-stan.org/misc/warnings.html#divergent-transitions-after-warmup
## to find out why this is a problem and how to eliminate them.
```

```
## Warning: Examine the pairs() plot to diagnose sampling problems
```

```
do.call("rbind", c(list(comp_fn(mod_seer)), res)) %>%
  mutate(knots = c("10,15,20",
                   unlist(lapply(knot_list, paste, collapse=",")))) %>%
  select(knots, rmf, looic) %>%
  knitr::kable(col.names=c("Additional knots",
                           "Restricted mean survival over 20 years","LOOIC"))
```

| Additional knots | Restricted mean survival over 20 years | LOOIC |
| --- | --- | --- |
| 10,15,20 | 5.77 (5.02, 6.56) | 1483 |
| 10,20 | 5.7 (4.98, 6.47) | 1506 |
| 20 | 5.77 (4.96, 6.54) | 1515 |
| 8,10,12,14,16,18,20 | 5.8 (5.1, 6.53) | 1509 |

# Population data in an additive hazards model

An alternative way of including external data is through additive
hazards (or relative survival), using the `backhaz` argument
to supply a data frame that describes the background hazard at all
times. This describes the population of the USA but matched to the trial
patients by age, sex and calendar year, and is supplied in the
`survextrap` package as `cetux_bh`.

```
head(cetux_bh)
```

```
##   time    hazard
## 1    0 0.0092656
## 2    1 0.0101710
## 3    2 0.0111254
## 4    3 0.0117602
## 5    4 0.0132862
## 6    5 0.0144244
```

The remaining data (trial and registry) are used to infer the excess
hazard for head and neck cancer patients. A model is fitted which
assumes the overall hazard for the trial and registry patients is the
sum of the background hazard (fixed from the data in
`backhaz`) and an unknown excess hazard to be estimated.

```
mod_seer_pop <- survextrap(Surv(years, d) ~ 1, data=control, mspline = mspline_seer,
                           external=cetux_seer, backhaz=cetux_bh, 
                           chains=chains,iter=iter,
                           prior_hscale=prior_hscale, prior_hsd = prior_hsd)
```

```
haz_seer <- hazard(mod_seer,tmax=20) %>% mutate(Model="Without population data")
haz_seer_pop <- hazard(mod_seer_pop,tmax=20) %>% mutate(Model="With population data")
haz_plot_reg_pop20 <- rbind(haz_seer, haz_seer_pop)

ggplot(haz_plot_reg_pop20, aes(x=t, y=median, col=Model, fill=Model)) + 
    geom_ribbon(aes(ymin=lower, ymax=upper), alpha=0.2, colour=NA) +
    geom_line() +
    geom_step(data=cetux_seer  %>% filter(stop < 20), 
              aes(x=start, y=haz), inherit.aes = FALSE) +
    geom_step(data=cetux_seer  %>% filter(stop < 20), 
              aes(x=start, y=haz_lower), col="gray60", lty=2, inherit.aes = FALSE) +
    geom_step(data=cetux_seer  %>% filter(stop < 20), 
              aes(x=start, y=haz_upper), col="gray60", lty=2, inherit.aes = FALSE) + 
    geom_step(data=cetux_bh %>% filter(time < 20), 
              aes(x=time, y=hazard), inherit.aes = FALSE, colour="darkgreen") +
    annotate(geom="text", x=10, y=0, label="Population mortality", colour="darkgreen", size=2) +
    theme(panel.grid.major = element_blank(), panel.grid.minor = element_blank()) +
    xlab("Years after diagnosis") + 
    ylab("Hazard") +
    scale_color_viridis(discrete=TRUE) + 
    geom_vline(xintercept = max(control$years)) +
    theme(legend.position = c(0.8, 0.9), 
          legend.background=element_blank()) + 
    labs(col=NULL, fill=NULL)
```

Including the population data does not make much difference to the
estimated hazards over 20 years after diagnosis.

The benefit of the population data in this example is to inform
extrapolations over a longer time horizon than the 25 year horizon of
the registry data. We now consider extrapolations up to 40 years. To
allow the hazard shape to change up to 40 years, we extend the spline
specification to include two further knots at 30 and 40 years, in
addition to the ones already placed at 10, 15 and 20 years.

Two models are then fitted, to show the impact of the population
data. The first includes the trial and registry data.

```
mspline40 <- mspline_spec(Surv(years, d) ~ treat, data=cetux, df=6, 
                          add_knots=c(10, 15, 20, 30, 40))
mod_seer40 <- survextrap(Surv(years, d) ~ 1, data=control, mspline = mspline40,
                         external=cetux_seer, 
                         chains=chains, iter=iter, 
                         prior_hscale=prior_hscale, prior_hsd = prior_hsd)
```

```
## Warning: There were 1 divergent transitions after warmup. See
## https://mc-stan.org/misc/warnings.html#divergent-transitions-after-warmup
## to find out why this is a problem and how to eliminate them.
```

```
## Warning: Examine the pairs() plot to diagnose sampling problems
```

The second includes the trial, registry and population data.

```
mod_seer_pop40 <- survextrap(Surv(years, d) ~ 1, data=control, mspline = mspline40,
                           external=cetux_seer, backhaz=cetux_bh,
                           chains=chains, iter=iter, 
                           prior_hscale=prior_hscale, prior_hsd = prior_hsd)
```

The population data “anchors” the extrapolation - the hazard is
constrained to be no less than that of the population, and the resulting
estimates become more precise. There is still a lot of uncertainty,
since (through the knots at 30 and 40 years) we have allowed for the
possibility that the excess hazard changes beyond the point (25-26
years) where we actually have data about it.

```
haz_seer40 <- hazard(mod_seer40,tmax=40) %>% 
    mutate(Model="With registry data")
haz_seer_pop40 <- hazard(mod_seer_pop40,tmax=40) %>% 
    mutate(Model="With registry and population data")
haz_plot_reg_pop <- rbind(haz_seer40, haz_seer_pop40)
ggplot(haz_plot_reg_pop, aes(x=t, y=median, col=Model, fill=Model)) + 
    ylim(0,0.5) +
    geom_ribbon(aes(ymin=lower, ymax=upper), alpha=0.2, colour=NA) +
    geom_line() +
    geom_step(data=cetux_seer, aes(x=start, y=haz), inherit.aes = FALSE) +
    geom_step(data=cetux_seer, aes(x=start, y=haz_lower), col="gray60", lty=2,
              inherit.aes = FALSE) +
    geom_step(data=cetux_seer, aes(x=start, y=haz_upper), col="gray60", lty=2,
              inherit.aes = FALSE) + 
    geom_step(data=cetux_bh %>% filter(time < 40), 
              aes(x=time, y=hazard), inherit.aes = FALSE, colour="darkgreen") +
    annotate(geom="text", x=33, y=0.1, label="Population mortality", colour="darkgreen", size=2) +
    theme(panel.grid.major = element_blank(), panel.grid.minor = element_blank()) +
    xlab("Years after diagnosis") + 
    ylab("Hazard") +
    scale_color_viridis(discrete=TRUE) + 
    theme(legend.position = c(0.4, 0.9), 
          legend.background=element_blank()) + 
    labs(col=NULL, fill=NULL) +
    geom_vline(xintercept = max(control$years))
```

# Mixture cure model

The extrapolation of the excess hazard is still fairly uncertain,
even when both registry and population data are included, since the data
informing the excess hazard finish at 25 years.

In many clinical applications, it is thought that the excess hazard
for a population with a disease will decrease over time, particularly as
people grow older and their all-cause mortality increases. We examine a
couple of ways to use this kind of judgement to improve survival
extrapolations using `survextrap`. The specific assumptions
used here may or may not be realistic for these patients: this case
study is intended as a demonstration of methods, rather than a full,
clinically-informed investigation of head and neck cancer survival.

One way of modelling a decreasing hazard is through a *mixture
cure* model. This can also be used to model a decreasing
*excess* hazard in an additive hazards model. This is simply
specified by setting `cure=TRUE` in the call to
`survextrap`.

```
mod_cure <- survextrap(Surv(years, d) ~ 1, data=control, mspline = mspline40,
                       external=cetux_seer, backhaz=cetux_bh, 
                       cure=TRUE, 
                       chains=chains, iter=iter,
                       prior_hscale=prior_hscale, prior_hsd = prior_hsd)
```

Optionally, a Beta prior could also be specified for the “cure
fraction” through
e.g. `survextrap(..., prior cure = p_beta(1,10))`, though the
default uniform prior is used here. The cure fraction is the proportion
of people who will never experience the event of interest. The survival
curve decreases to an asymptote at the cure fraction, hence (informally)
the cure fraction can sometimes be inferred from data by examining the
survival curve. Often however it is weakly informed by data.

In this example, the excess hazard for head and neck cancer patients
is still substantial by the end of the registry data (25 years), so
there is little evidence of “cure” from the data. Therefore the
extrapolations from the fitted cure model are very uncertain (see below), and largely overlap with those from
the equivalent model for the same data where cure was not assumed (see above). In addition, the cure model makes a
strong parametric assumption for the shape of how the hazard decreases
over time.

# Elicited data to inform long-term extrapolations

An alternative way to build in judgements about longer-term survival
is through expert elicitation, as discussed in the priors vignette.

Here, suppose we are confident that by 40 years, the survival of head
and neck cancer patients will be similar to that of the general
population. Instead of using a parametric cure model, we can create an
artificial external dataset that contains the same information as our
judgement. Here the annual population survival probability is computed
from the hazard stored in `cetux_bh`, and stored in
`pops`.

```
cetux_bh <- cetux_bh %>% 
  mutate(surv1 = exp(-hazard*1))
pops <- cetux_bh$surv1[39]
```

A `Beta(n*pops, n(1-pops))` distribution for any
`n` will have a mean of `pops`, here 0.72. We then
set `n` to control the amount of uncertainty around this
prior guess.

```
qbeta(c(0.025, 0.5, 0.975), 1000*pops, 1000*(1-pops))
```

```
## [1] 0.6961908 0.7244400 0.7515403
```

The information in this Beta prior is equivalent to having observed
`1000*pops` survivors out of 1000 people. This gives a
numerator and denominator `n` and `r` for survival
between `start=39` and `stop=40` years. We use
these to construct a dataset in the format needed by the
`external` argument to `survextrap`.

```
data_elic <- data.frame(
  start=39, stop=40,
  n = 1000, 
  r = round(1000*pops),
  treat = "Control"
)
```

This can then be joined to the registry data `cetux_seer`,
to give an external dataset to supply to `survextrap`.

```
pop_cured <- 
  cetux_seer %>% full_join(data_elic, by = join_by(start, stop, r, n, treat))
mod_cure_elic <- survextrap(Surv(years, d) ~ 1, data=control, mspline=mspline40, 
                            external=pop_cured, backhaz=cetux_bh, 
                            chains=chains, iter=iter,
                            prior_hscale=prior_hscale, prior_hsd = prior_hsd)
```

The extrapolations of excess hazard from this model are clearly informed
by the elicited survival probability, and pulled closer to the
population data. If we had expressed more confidence in the prior guess
(through a bigger `n`) the posterior estimates of the hazard
function from the model would have been pulled even closer to the
population data. 


```
haz_cure <- hazard(mod_cure, tmax=40) %>% mutate(Model="Mixture cure model")
haz_cure_elic <- hazard(mod_cure_elic, tmax=40) %>% mutate(Model="Elicited survival")
haz_plot_cure <- rbind(haz_cure, haz_cure_elic)

ggplot(haz_plot_cure, aes(x=t, y=median, col=Model, fill=Model)) + 
    ylim(0,0.5) +
    geom_ribbon(aes(ymin=lower, ymax=upper), alpha=0.2, colour=NA) +
    geom_line() +
    geom_step(data=cetux_seer, aes(x=start, y=haz), inherit.aes = FALSE) +
    geom_step(data=cetux_seer, aes(x=start, y=haz_lower), col="gray60", lty=2,
              inherit.aes = FALSE) +
    geom_step(data=cetux_seer, aes(x=start, y=haz_upper), col="gray60", lty=2,
              inherit.aes = FALSE) + 
    geom_step(data=cetux_bh %>% filter(time < 40), 
              aes(x=time, y=hazard), inherit.aes = FALSE, col="darkgreen") +
    annotate(geom="text", x=20, y=0, label="Population mortality", col="darkgreen", size=2) +
    theme(panel.grid.major = element_blank(), panel.grid.minor = element_blank()) +
    xlab("Years after diagnosis") + 
    ylab("Hazard") +
    scale_color_viridis(discrete=TRUE) + 
    geom_vline(xintercept = max(control$years)) +
    theme(legend.position = c(0.4, 0.9), 
          legend.background=element_blank()) + 
    labs(col=NULL, fill=NULL)
```

# Table of mean survival times

A table of results for the paper is now constructed and formatted. We
compare restricted mean survival times, over various time horizons,
estimated from the various models fitted to the control group data.

The models are categorised by what data are included, and what
assumptions are made about survival beyond the data.

```
ni <- 4000

rcon <- rmst(mod_con, t=5, niter=ni) %>% 
  mutate(data="Trial",extrap="No extrapolation")
rcon20 <- rmst(mod_con, t=20, niter=ni) %>% 
  mutate(data="Trial", extrap="Uncertain hazard")
rcon520 <- rmst(mod_con5, t=20, niter=ni) %>% 
  mutate(data="Trial", extrap="Constant hazard")
rreg20 <- rmst(mod_seer, t=20, niter=ni) %>% 
  mutate(data="Trial,registry",extrap="No extrapolation")
rreg40 <- rmst(mod_seer40, t=40, niter=ni)  %>% 
  mutate(data="Trial,registry", extrap="Uncertain hazard")
rpop40 <- rmst(mod_seer_pop40, t=40, niter=ni) %>%
  mutate(data="Trial,registry,population",extrap="Uncertain excess hazard")
rcure40 <- rmst(mod_cure, t=40, niter=ni) %>% 
  mutate(data="Trial,registry,population", 
         extrap="Mixture cure")
relic40 <- rmst(mod_cure_elic, t=40, niter=ni) %>%
  mutate(data="Trial,registry,population", 
         extrap="Elicited survival")

modlabs <- c("(1a)", "(1a)", "(1b)", "(1c)", "(1d)","(1e)", "(1f)", "(1g)")

rs <- list(rcon, rcon20, rcon520, rreg20, rreg40, rpop40, rcure40, relic40) %>%
  purrr::reduce(full_join, by = join_by(variable, t, median, `2.5%`, `97.5%`, data, extrap)) %>%
  mutate(modlabs=modlabs,
         rmst = sprintf("%s (%s, %s)", round(median,2), 
                        round(`2.5%`,2), round(`97.5%`,2))) %>%
  select(modlabs, data, extrap, t, rmst)
knitr::kable(rs, col.names=c("Model", "Observed data","Extrapolation assumptions",
                             "Time horizon","Restricted mean survival"))
```

| Model | Observed data | Extrapolation assumptions | Time horizon | Restricted mean survival |
| --- | --- | --- | --- | --- |
| (1a) | Trial | No extrapolation | 5 | 2.88 (2.63, 3.13) |
| (1a) | Trial | Uncertain hazard | 20 | 5.11 (3.77, 7.14) |
| (1b) | Trial | Constant hazard | 20 | 5.12 (4.01, 6.71) |
| (1c) | Trial,registry | No extrapolation | 20 | 5.76 (5.04, 6.57) |
| (1d) | Trial,registry | Uncertain hazard | 40 | 6.2 (5.35, 7.12) |
| (1e) | Trial,registry,population | Uncertain excess hazard | 40 | 6.22 (5.36, 7.13) |
| (1f) | Trial,registry,population | Mixture cure | 40 | 6.27 (5.39, 7.22) |
| (1g) | Trial,registry,population | Elicited survival | 40 | 6.26 (5.37, 7.17) |

The following code formats the table in LaTeX format (for the
paper).

```
write.table(rs, sep="  &  ", eol="\\\\\n", quote=FALSE, row.names = FALSE)
```

```
## modlabs  &  data  &  extrap  &  t  &  rmst\\
## (1a)  &  Trial  &  No extrapolation  &  5  &  2.88 (2.63, 3.13)\\
## (1a)  &  Trial  &  Uncertain hazard  &  20  &  5.11 (3.77, 7.14)\\
## (1b)  &  Trial  &  Constant hazard  &  20  &  5.12 (4.01, 6.71)\\
## (1c)  &  Trial,registry  &  No extrapolation  &  20  &  5.76 (5.04, 6.57)\\
## (1d)  &  Trial,registry  &  Uncertain hazard  &  40  &  6.2 (5.35, 7.12)\\
## (1e)  &  Trial,registry,population  &  Uncertain excess hazard  &  40  &  6.22 (5.36, 7.13)\\
## (1f)  &  Trial,registry,population  &  Mixture cure  &  40  &  6.27 (5.39, 7.22)\\
## (1g)  &  Trial,registry,population  &  Elicited survival  &  40  &  6.26 (5.37, 7.17)\\
```

# Waning models for long-term treatment effects

Finally, we estimate the effect of treatment with cetuximab on
long-term survival. We extend two of the models with external data
(fitted earlier to just the control group) to include a treatment effect
and fit them to the full trial data `cetux`. Firstly, the
model with trial and registry data, and secondly, the model with all
three data sources (trial, registry and population, but no “cure”
assumption). The proportional hazards model is assumed, which was
adequate to describe the short-term data.

```
mod_full_reg <- survextrap(Surv(years, d) ~ treat, data=cetux, mspline = mspline40,
                           external=cetux_seer, 
                           chains=chains, iter=iter, 
                           prior_hscale=prior_hscale, prior_hsd = prior_hsd, 
                           prior_loghr = prior_loghr)
mod_full_pop <- survextrap(Surv(years, d) ~ treat, data=cetux, mspline = mspline40,
                           external=cetux_seer, backhaz=cetux_bh,
                           chains=chains, iter=iter, 
                           prior_hscale=prior_hscale, prior_hsd = prior_hsd, 
                           prior_loghr = prior_loghr)
```

The restricted mean survival for the control group, and the increase
in the restricted mean survival given by cetuximab, is estimated under
various “treatment effect waning” assumptions. Essentially these assume
that the log hazard ratio for a “treated” group decreases linearly over
some time period from the “treated” estimate to the “control” estimate.
This is done by mixing the posteriors of treated and control estimates
obtained from the same model, in this case `mod_full_pop`
(see the methods vignette).

The calls to `rmst` and `irmst` are modified to
include a `wane_period` (vector defining the start and end of
the waning period). We also supply `newdata` and
`newdata0`, which indicate respectively what covariate values
in the model correspond to “treated” and “control” groups.

```
nd <- data.frame(treat=c("Cetuximab"))
nd0 <- data.frame(treat=c("Control"))
ind <- data.frame(treat=c("Control","Cetuximab"))
ind0 <- data.frame(treat=c("Control","Control"))
tmax <- max(cetux$years)

ni <- 1000

## 20 year absolute survival
rmnwr <- rmst(mod_full_reg, t=20, niter=ni) %>% mutate(wane=0)
rmnw <- rmst(mod_full_pop, t=20, niter=ni) %>% mutate(wane=0)
rmw6 <- rmst(mod_full_pop, t=20, niter=ni, newdata=nd, newdata0=nd0, 
             wane_period = c(tmax, 6)) %>% mutate(wane=6)
rmw20 <- rmst(mod_full_pop, t=20, niter=ni, newdata=nd, newdata0=nd0, 
              wane_period = c(tmax, 20)) %>% mutate(wane=20)

## 20 year incremental survival for cetuximab
irmnwr <- irmst(mod_full_reg, t=20, niter=ni) %>% mutate(wane=0)
irmnw <- irmst(mod_full_pop, t=20, niter=ni) %>% mutate(wane=0)
irmw6 <- irmst(mod_full_pop, t=20, niter=ni, newdata = ind, newdata0 = ind0, 
               wane_period = c(tmax,6), wane_nt = 5) %>% mutate(wane=6)
irmw20 <- irmst(mod_full_pop, t=20, niter=ni, newdata = ind, newdata0 = ind0, 
                wane_period = c(tmax,20), wane_nt = 5) %>% mutate(wane=20)

rmd <- rbind(rmnwr, rmnw, rmw6, rmw20) %>% 
    filter(treat=="Control") %>%
    rename(rm="median",rmlower="2.5%",rmupper="97.5%") %>%
    mutate(data=c("reg","pop"),
           rmf = sprintf("%s (%s, %s)", round(rm,2), 
                         round(rmlower,2), round(rmupper,2))) %>%
    select(data, wane, rmf) 
irmd <- rbind(irmnwr, irmnw, irmw6, irmw20) %>% 
    rename(irm="median",irmlower="2.5%",irmupper="97.5%") %>%
    mutate(data=c("reg","pop","pop","pop"),
           irmf = sprintf("%s (%s, %s)", round(irm,2), 
                          round(irmlower,2), round(irmupper,2))) %>%
    select(data, wane, irmf)
res_wane <- rmd %>% 
    select(-wane) %>%
    right_join(irmd, by=c("data")) %>%
  mutate(model=c("(2d) No waning","(2e) No waning",
                 "(2e) 5 to 6 years", "(2e) 5 to 20 years"),
           looic = "",
           rmf = tidyr::replace_na(rmf, "")) %>%
    select(model, rmf, irmf, looic)
knitr::kable(res_wane %>%
             select(-looic) %>%
             mutate(data = c("Trial,registry",rep("Trial,registry,population",3))) %>%
             relocate(data),
             col.names=c("Data","Model","Restricted mean survival",
                         "Increase in restricted mean survival"))
```

| Data | Model | Restricted mean survival | Increase in restricted mean survival |
| --- | --- | --- | --- |
| Trial,registry | (2d) No waning | 5.82 (5.07, 6.62) | 1.23 (-0.34, 2.86) |
| Trial,registry,population | (2e) No waning | 5.89 (5.11, 6.65) | 1.08 (-0.4, 2.61) |
| Trial,registry,population | (2e) 5 to 6 years | 5.89 (5.11, 6.65) | 0.81 (-0.3, 1.92) |
| Trial,registry,population | (2e) 5 to 20 years | 5.89 (5.11, 6.65) | 1.02 (-0.38, 2.47) |

Table in LaTeX format (for the paper).

```
write.table(res_wane, eol="\\\\\n", sep="  &  ", row.names = FALSE, quote=FALSE)
```

```
## model  &  rmf  &  irmf  &  looic\\
## (2d) No waning  &  5.82 (5.07, 6.62)  &  1.23 (-0.34, 2.86)  &  \\
## (2e) No waning  &  5.89 (5.11, 6.65)  &  1.08 (-0.4, 2.61)  &  \\
## (2e) 5 to 6 years  &  5.89 (5.11, 6.65)  &  0.81 (-0.3, 1.92)  &  \\
## (2e) 5 to 20 years  &  5.89 (5.11, 6.65)  &  1.02 (-0.38, 2.47)  &  \\
```

Using all three data sources, with no waning, the incremental
restricted mean survival over 20 years is estimated as 1.08 (-0.4,
2.61). This reduces to 1.02 (-0.38, 2.47) when waning is applied
gradually from 6 to 20 years, and even further to 0.81 (-0.3, 1.92) when
the effect is assumed to wane rapidly from 5 to 6 years.
